# Anti-Integrin Therapy for Multiple Sclerosis

**DOI:** 10.1155/2012/357101

**Published:** 2012-12-17

**Authors:** Eiji Kawamoto, Susumu Nakahashi, Takayuki Okamoto, Hiroshi Imai, Motomu Shimaoka

**Affiliations:** ^1^Emergency and Critical Care Center, Mie University Hospital, 2-174 Edobashi, Tsu 514-8507, Japan; ^2^Departments of Molecular Pathobiology and Cell Adhesion Biology, Mie University Graduate School of Medicine, 2-174 Edobashi, Tsu 514-8507, Japan

## Abstract

Integrins are the foremost family of cell adhesion molecules that regulate immune cell trafficking in health and diseases. Integrin alpha4 mediates organ-specific migration of immune cells to the inflamed brain, thereby playing the critical role in the pathogenesis of multiple sclerosis. Anti-alpha4 integrin therapy aiming to block infiltration of autoreactive lymphocytes to the inflamed brain has been validated in several clinical trials for the treatment of multiple sclerosis. This paper provides readers with an overview of the molecular and structural bases of integrin activation as well as rationale for using anti-alpha4 integrin therapy for multiple sclerosis and then chronicles the rise and fall of this treatment strategy using natalizumab, a humanized anti-alpha4 integrin.

## 1. Introduction

Multiple sclerosis (MS) is a devastating autoimmune disease that is characterized by inflammation in the brain and spinal cord that damages the myelin sheath, thereby causing demyelination of neurons [1–3]. Demyelination impairs neuronal signal transmission, which in turn results in various physical and cognitive disabilities such as sensory disorder, motor dysfunction, optic neuritis, and coordination problems. The disease worsens in many patients during relapses, and effective treatments to block the relapses remain limited. In this way, multiple sclerosis imposes a substantial economic burden across developed countries worldwide [4]. 

Infiltration of the brain by autoreactive immune cells that originate in the peripheral circulation plays a central role in the pathogenesis of inflammation in MS [3, 5]. During the last decade, significant progress has been made in understanding how pathogenic leukocytes migrate from the periphery to the central nervous system (CNS). The critical roles played by a specific cell adhesion molecule, alpha4 integrin, in leukocyte-endothelial cell interactions at the blood brain barrier have been studied extensively in animal models, as well as in patients in clinical trials [6]. It is worth noting that natalizumab (Tysabri), a blocking antibody to alpha4 integrin, was approved by the FDA in 2004 for the treatment of relapsing multiple sclerosis patients. This paper describes the molecular and structural bases for using anti-integrin therapy for multiple sclerosis and then discusses both the promise and problems with this treatment strategy including the importance of clinical pharmacovigilance in the risk management of natalizumab treatment.

## 2. Integrins Regulate Leukocyte-Endothelial Interactions

### 2.1. Leukocyte Integrins

Alpha4 integrin is among 24 integrin families of cell-adhesion molecules containing noncovalently-associated alpha and beta subunits [7–10]. Eighteen different integrin alpha subunits and eight different beta subunits have been reported to date in vertebrates, forming at least 24 alpha/beta heterodimers. These varied formations suggest that integrins constitute the most structurally and functionally diverse family of cell-adhesion molecules yet known. Integrins mediate cell-cell and cell-extracellular matrix interactions over a wide range of biological contexts. Integrins support force-resistant stable firm adhesion as well as the dynamic adhesive interactions observed in cellular polarization and cell migration. Integrins play a crucial role in many physiological processes including tissue morphogenesis, inflammation, wound healing, and regulation of cell growth and differentiation. 

### 2.2. Integrin Activation

What makes integrins very unique in many cell adhesion molecules is their ability to transmit signals across the plasma membrane bidirectionally [7]. Activation of other receptors such as chemokine-receptors or T-cell receptors elicits signaling pathways that culminate to the binding of key intracellular proteins talins and kindlins to integrin cytoplasmic domains, thereby inducing the separation of integrin cytoplasmic domains that otherwise associate each other [11]. This triggers global conformational conversion to the high-affinity form that enhances the activity of the extracellular headpiece for ligand binding (inside-out signaling). Conversely, the binding of ligand to integrin extracellular domains stabilizes the high-affinity conformation, thereby facilitating the separation of the integrin cytoplasmic tails that initiates intracellular signaling (outside-in signaling). Such bidirectional signaling supports the dynamic and reversible transformation of integrins between nonadhesive and adhesive states and thereby plays a critical role in the regulation of cell adhesion and migration.

### 2.3. Alpha4 Integrin

The adhesive and signaling activities carried out by integrins are vital to many of the cell-cell and cell-extracellular matrix interactions involved in immune responses [12]. Along with beta2 integrins, alpha4 integrin plays a critical role in their adhesive interactions with endothelial cells during migration to lymphoid organs and extravasation to sites of inflammation [13–15]. Alpha4 integrin subunit pairs with the beta1 and beta7, thereby constituting integrin alpha4beta1 (a.k.a. very late antigen-4, VLA-4) and alpha4beta7 (a.k.a. lymphocyte Peyer's patch adhesion molecules; LPAM-1) receptors. Alpha4beta1 binds to the major endothelial ligand VCAM-1 and extracellular matrix ligand fibronectin deposited in inflamed tissues, while alpha4beta7 binds to MAdCAM-1 preferentially expressed in the gut.

A beta2 integrin alphaLbeta2 (a.k.a. leukocyte function-associated antigen-1: LFA-1) that binds to ICAM-1 on endothelial cells critically regulates adhesive leukocyte interactions on the luminal surface of the vasculature during extravasation to inflamed tissues, as well as during normal recirculation through the lymphoid tissues of lymphocytes [12, 16]. In contrast alpha4 integrins play crucial roles in tissue-specific leukocyte trafficking to the inflamed brain (alpha4beta1) and to the inflamed gut (alpha4beta7). Alpha4 integrins also regulate hematopoietic stem cell trafficking and retention in the bone marrow [15]. 

Alpha4 integrin is predominantly in hematologic cells in adults; however, it is highly expressed in the heart (i.e., pericardium) during early development in embryo. This explains why nonconditional alpha4 integrin knockout mice exhibited embryonic lethality [17]. This might raise a potential clinical concern of using anti-alpha4 integrin therapy for pregnant patients.

### 2.4. Genetic Defects of Integrin Functionality

The physiologic importance of the ability to upregulate leukocyte integrins is illustrated by two rare genetic disorders: leukocyte adhesion deficiency type I (LAD-I) and type III (LAD-III). LAD-I is caused by loss-of-function mutations in the beta2 integrin subunit that result in the absence, or severely reduced expression, of all beta2 integrin heterodimers on the cell surface of leukocytes [18, 19]. LAD-I patients suffer from recurrent and often life-threatening bacterial infections and from impaired wound healing, since beta2 integrins are important for host defenses against microorganisms. Neutrophils from LAD-I patients show a markedly reduced capacity to adhere to endothelial cells or to migrate to sites of inflammation. LAD-I lymphocytes exhibit impaired function in antigen- and mitogen-induced proliferation, antibody-dependent killing, and T cell-dependent antibody production. 

Not only beta2 integrins, but also alpha4 integrins are severely affected in LAD-III that is caused by a genetic defect in kindlin-3, a cytoskeletal protein that activates integrins by binding to integrin beta cytoplasmic tails. LAD-III patients manifest not only an increased susceptibility to bacterial infections, such as occurs with LAD-I, but also platelet dysfunction [20]. The latter can be observed in Glanzmann thrombasthenia, where one finds a lack of integrin alphaIIbbeta3 expression or functionality. Despite normal levels of integrin expression, these same LAD-III leukocytes fail to upregulate alphaLbeta2 and alpha4beta1 adhesiveness in response to chemokines and/or the other chemoattractants that activate GPCR signaling.

## 3. Integrins in Leukocyte-Endothelial Interactions

### 3.1. Steps in Cell Adhesion Cascades

Persistent accumulation of leukocytes is a hallmark of the chronic inflammation observed in the affected tissues of autoimmune diseases [21, 22]. For leukocytes to accumulate within inflamed tissues, they must interact with and subsequently pass an endothelial monolayer lining on the inner surface of the vasculature. The leukocyte-endothelial interactions leading to extravasation are regulated by a sequence of multiple steps involving adhesion molecules and chemokine signaling. At inflammatory sites, circulating leukocytes that flow in blood vessels start to tether and roll along endothelial cells via selectins and their ligands. This rolling interaction serves to slow down leukocytes and place them in proximity to the inflamed endothelial cells, thereby enabling these cells to efficiently scan the endothelial surface for available chemokines. While rolling, leukocytes encounter chemokines and become activated via chemokine receptors present on the leukocytes. Chemokine signaling elicits an intracellular signaling cascade that eventually impinges on integrin cytoplasmic domains, thereby triggering integrin activation. Upon activation, global conformational changes occur that rapidly convert a low-affinity latent integrin into a high-affinity ligand-competent state. In this way, the high-affinity integrin mediates the rapid arrest of rolling leukocytes and shear-resistant firm adhesion at inflamed endothelial cells. 

The cascade of leukocyte-endothelial cell interactions was originally thought to contain three steps (i.e., rolling by selectins, activation by chemokines, and upregulation of integrin affinity) before transmigration of leukocytes across the endothelial barrier could occur. A crawling step has recently been added to this cascade. Specifically, leukocytes crawl along the endothelial surface from the point of arrest to that of transmigration [23, 24]. The dynamic regulation of integrin affinity plays a critical role in supporting such leukocyte crawling [25]. 

### 3.2. Alternative Routes and Steps of Transendothelial Migration

During transendothelial migration (TEM), leukocytes proceed through endothelial cells via two distinct routes, either paracellular or transcellular [26]. In the former, leukocytes transmigrate in between adjacent endothelial cells, which usually form tightly sealed junctions, thereby leaving no space between them. During paracellular TEM, the junctions between endothelial cells are dynamically disassembled, thereby creating a gap for a leukocyte to pass through. This gap is closed as soon as the trailing edge of the leukocyte passes beyond it. In contrast, during transcellular TEM, a leukocyte transmigrates through a single endothelial cell. A pore is formed for a leukocyte to move through. The pore formation that develops during transcellular TEM is thought to be mediated by a dynamic remodeling of the endothelial cell-rich plasma membrane that involves vimentin as well as caveolae or vesiculovacuolar organelles [27, 28]. How leukocytes decide to take one of the two routes for TEM and find a site for TEM remains to be elucidated. To probe the surface of an endothelial cell for a “hot spot” to transmigrate, crawling leukocytes might use podosomes, actin-rich finger-like structures that resemble the invadosomes seen in certain invasive cancer cells [29]. 

Pericytes are located on the basolateral side of vascular endothelial cells, wherein they surround the endothelial monolayers. Pericytes were thought to play merely a house-keeping and scaffolding role to support vascular endothelial cells; however, a recent investigation has revealed that pericytes in fact play a novel role in regulating leukocyte migration into interstitial spaces. Transmigrated leukocytes have been found to crawl in the perivascular space between the endothelial basement membrane and underlying pericytes before they enter an interstitial space [30]. Before entering such an interstitial space, leukocytes crawling the perivascular space must proceed through a gap in between adjacent pericytes. Of note, in the presence of inflammation, the gaps in between adjacent pericytes are enlarged, thereby promoting the entry of leukocytes into inflamed tissues. This observation suggests that pericytes play a novel gatekeeper role in regulating leukocyte entry into the interstitial spaces.

### 3.3. Tissue-Specific Homing

Organ-specific homing of leukocytes is made possible primarily by unique combinations of cell-adhesion molecules and chemokine receptors [31, 32]. Naïve lymphocytes recirculate between the blood stream and peripheral lymphoid tissues, thereby patrolling the body for microorganisms and transformed cells. To enter peripheral lymphoid tissues, naive T lymphocytes roll via L-selectin, are activated via a chemokine receptor CCR7, and then become arrested via the integrin LFA-1 on high endothelial values in peripheral lymph nodes. Upon activation, L-selectin shedding occurs, and the resulting effector T lymphocytes lose their ability to recirculate through peripheral lymph nodes. Depending on how and where they are activated, effector T lymphocytes increase the cell-surface expression of different integrins and chemokine receptors, thereby acquiring distinct organ tropisms [31]. For example, skin-tropic effector T cells, which are responsible for psoriasis pathogenesis, upregulate in integrin alphaLbeta2, as well as in the chemokine receptors CCR4 and CCR10 along with P-selectin ligand. Gut-tropic effector T cells, which play a pathogenic role in inflammatory bowel diseases, upregulate in integrin alpha4beta7 and the chemokine receptor CCR9. Brain-tropic effector T cells, which are responsible for the pathogenesis of multiple sclerosis, upregulate in integrin alpha4beta1.

## 4. Rise and Fall of Anti-Integrin Therapies

### 4.1. Rise of Anti-Alpha4 Integrin Therapy with Natalizumab

The concept that the organ specificity of autoreactive effector T-cell homing relies on specific integrins led to the development of anti-integrin therapies for the treatment of autoimmune diseases including multiple sclerosis. A seminal *in vivo* experiment conducted by Yednock et al. showed that a blocking antibody to integrin alpha4beta1 prevented the infiltration of leukocytes to the brain, thereby inhibiting the development of experimental autoimmune encephalomyelitis (EAE), an established mouse model for multiple sclerosis [33]. Capitalizing on this work, as well as on other *in vivo* studies [34–36], two biotech companies, the Cambridge-based Biogen and the San Francisco-based Elan, codeveloped the humanized monoclonal antibody natalizumab (Tysabri, a.k.a. Antegren) to the integrin alpha4 subunit.

The first randomized, double-blind, placebo-controlled trial that showed natalizumab to have some clinical efficacy in multiple sclerosis was performed in the UK [37]. In this small trial, which involved 72 patients with active relapsing-remitting and secondary progressive MS, a short-term effect stemming from administration of natalizumab was investigated. Specifically, patients were administered only two infusions of the antibody one month apart. The natalizumab treatment effectively reduced the appearance of new active lesions, as shown in MRI brain scans. Although the treatment had no significant impact on the number of acute MS exacerbations, this study was not designed to determine the effects of treatment on the rate of relapse.

A subsequent randomized, double-blind, placebo-controlled trial involving 213 patients was performed in 26 hospitals across the USA, Canada, and UK [38]. The trial studied long-term (i.e., 6 months) effects of natalizumab treatment in patients with relapsing MS. Natalizumab treatment not only reduced the number of new MRI brain lesions, but also improved clinical outcomes, that is, decreased the number of relapses.

Capitalizing on these pioneering clinical trials, two randomized, multicentre, placebo-controlled, double-blind trials, AFFIM and SENTINEL, were subsequently carried out. The AFFIRM (Natalizumab Safety and Efficacy in Relapsing and Remitting Multiple Sclerosis) study was designed to evaluate the effects of the antibody on the progression of disability and the rate of clinical relapses [39]. In this study, involving 942 people across North America, Europe, and Australasia, patients were treated with either natalizumab or a placebo for more than 2 years. The two-year data showed that compared with the placebo, natalizumab treatment significantly reduced the rate of clinical relapses and increased the duration of relapse-free periods.

The SENTINEL (Safety and Efficacy of Natalizumab in Combination with Interferon Beta-1a in Patients with Relapsing Remitting Multiple Sclerosis) study was designed to evaluate the effects of adding natalizumab to a treatment regimen with interferon beta-1a [40]. In the SENTINEL study, which involved 1,171 people who continued to experience relapse(s) despite taking interferon beta-1a, patients received either natalizumab or a placebo, in addition to interferon beta-1a, for more than 2 years. Compared with interferon beta-1a plus placebo, the addition of natalizumab to interferon beta-1a significantly reduced the risk of disability progression, as well as the rate of clinical relapse. In this way, anti-integrin therapy with natalizumab appeared to be a magic bullet that would revolutionize medical care for multiple sclerosis patients. 

### 4.2. Downfall and Return of Natalizumab

Natalizumab was approved in 2004 through the FDA's accelerated First Track Program; however, the antibody soon suffered a major setback when two cases from the MS patients in the SENTINEL study and another from Crohn's disease (CD) patients treated with natalizumab developed progressive multifocal leukoencephalopathy (PML) [41–44]. PML represents a deadly and often fatal opportunistic infection that severely damages brain tissues. PML is caused by the reactivation of a latent JC virus infection during immunosuppression [45]. Alpha4 integrin appears to be important for maintaining anti-JC virus immunity by regulating the trafficking and retention of JC virus-specific memory/effector T cells [46].

After two deaths (one in MS and another in CD) of the three PML cases that resulted from natalizumab treatment, Biogen Idec voluntarily withdrew the antibody from the market in February of 2005, 4 months after accelerated FDA approval. However, in June of 2006, after comprehensively reviewing all patients exposed to natalizumab during clinical trials and carefully considering both the risk of fatal PML and the benefits of the treatment, the FDA advisory committee reapproved natalizumab for the treatment of relapsing MS patients [47].

The comprehensive reviews of the patients in postmarketing surveillance have identified three PML risks associated with natalizumab including positive serum anti-JC virus antibody titers that serve as a robust biomarker [48], prior or current immunosuppressive therapy, and duration of natalizumab treatment (especially duration longer than 2 years) [49, 50].

Natalizumab is currently regarded as the most potent treatment for relapsing-remitting MS; however, the PML risk restricts its indications [49, 50]. In the USA, natalizumab is generally recommended only for patients who have had a suboptimal response to standard treatment such as interferon-beta or glatiramer acetate, cannot tolerate one of the standard treatments, or have a poor prognosis. To prevent and monitor for the occurrence of PML, natalizumab is available in the USA only through an enhanced patient safety program termed the TOUCH (Tysabri Outreach Unified Commitment to Health) prescribing program. TOUCH prescribing program is to authorize patients, prescribers, pharmacies, and infusion sites, thereby serving as an important component of a global pharmacovigilance plan for mitigating the PML risk associated with natalizumab. 

## 5. Concluding Remarks

Alpha4 integrin-mediated trafficking of pathogenic effector T cells to the brain has been a validated therapeutic target for the treatment of MS. Building on the success of natalizumab, which validated this therapeutic target in patients, the effectiveness of firategrast, an orally active small-molecule antagonist to integrin alpha4 that is manufactured by GSK, has been investigated in patients with MS in a multicentre, phase 2, randomized, double-blind, placebo-controlled, dose-ranging study involving 343 patients [51]. Oral administration of firategrast has been shown to be well tolerated and to reduce the number of active regions in the brain, as evident in MRI scans. Importantly, no cases of PML have been seen in patients receiving firategrast. We await further results from phase 3 clinical trials with firategrast. 
